# Disodium tris­(dioxidomolybdenum) bis­(diarsenate)

**DOI:** 10.1107/S1600536812010069

**Published:** 2012-03-14

**Authors:** Raja Jouini, Mohamed Faouzi Zid, Ahmed Driss

**Affiliations:** a Laboratoire de Matériaux et Cristallochimie, Faculté des Sciences de Tunis, Université de Tunis ElManar, 2092 ElManar II Tunis, Tunisia

## Abstract

The asymmetric unit of the title compound, Na_2_(MoO_2_)_3_(As_2_O_7_)_2_, is composed of two cyclic MoAs_2_O_11_ units and an MoO_6_ corner-sharing octa­hedron. The anionic framework can be decomposed into two types of layers, *viz.* MoO_2_As_2_O_7_ and Mo_2_As_2_O_14_, which use mixed Mo—O—As and As—O—Mo bridges to achieve a new three-dimensional structure with two types of large channels in which the Na^+^ cations are located. Two O atoms are disordered and are located in two positions close to their initial positions with occupancy ratios of 0.612 (17):0.388 (17) and 0.703 (12):0.298 (12).

## Related literature

For background to the search for new materials with open structures, see: Kierkegaard & Westerlund (1964 ▶); Lii *et al.* (1987 ▶); Guesdon *et al.* (1994 ▶); Masquelier *et al.* (1995 ▶). In these materials, the association of *X*O_4_ (*X* = P, As) tetra­hedra and *M*O_6_ (*M* = transition metal) octa­hedra forms covalent hybrid structures that delimit tunnels, see: Linnros (1970 ▶); Hammond & Barbier (1996 ▶). For details of the preparation, see: Zid & Jouini (1996*a* ▶,*b* ▶); Zid *et al.* (1997 ▶, 1998 ▶); Hajji *et al.* (2004 ▶); Hajji & Zid (2006 ▶); Ben Hlila *et al.* (2009 ▶). For related structures, see: Averbuch-Pouchot (1988 ▶, 1989 ▶); Zid *et al.* (2003 ▶); Lii & Wang (1989 ▶); Benhamada *et al.* (1992 ▶). For the properties of related compounds, see: Marzouki *et al.* (2010 ▶); Ouerfelli *et al.* (2007 ▶). For background to the bond-valence method, see: Brown & Altermatt (1985 ▶).

## Experimental

### 

#### Crystal data

Na_2_(MoO_2_)_3_(As_2_O_7_)_2_
*M* *_r_* = 953.48
Monoclinic, 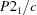

*a* = 14.571 (3) Å
*b* = 12.580 (2) Å
*c* = 9.258 (2) Å
β = 94.51 (2)°
*V* = 1691.9 (6) Å^3^
*Z* = 4
Mo *K*α radiation
μ = 10.11 mm^−1^
*T* = 298 K
0.26 × 0.18 × 0.14 mm

#### Data collection

Enraf–Nonius CAD-4 diffractometer
Absorption correction: ψ scan (North *et al.*, 1968 ▶) *T*_min_ = 0.133, *T*_max_ = 0.246
4271 measured reflections
3676 independent reflections
3128 reflections with *I* > 2σ(*I*)
*R*_int_ = 0.022
2 standard reflections every 120 min intensity decay: 1.1%

#### Refinement

*R*[*F* ^2^ > 2σ(*F* ^2^)] = 0.025
*wR*(*F* ^2^) = 0.060
*S* = 1.06
3676 reflections
273 parameters
2 restraints
Δρ_max_ = 0.98 e Å^−3^
Δρ_min_ = −0.75 e Å^−3^

### 

Data collection: *CAD-4 EXPRESS* (Duisenberg, 1992 ▶; Macíček & Yordanov, 1992 ▶); cell refinement: *CAD-4 EXPRESS*; data reduction: *XCAD4* (Harms & Wocadlo, 1995 ▶); program(s) used to solve structure: *SHELXS97* (Sheldrick, 2008 ▶); program(s) used to refine structure: *SHELXL97* (Sheldrick, 2008 ▶); molecular graphics: *DIAMOND* (Brandenburg, 1998 ▶); software used to prepare material for publication: *WinGX* (Farrugia, 1999 ▶).

## Supplementary Material

Crystal structure: contains datablock(s) I, global. DOI: 10.1107/S1600536812010069/fj2524sup1.cif

Structure factors: contains datablock(s) I. DOI: 10.1107/S1600536812010069/fj2524Isup2.hkl

Additional supplementary materials:  crystallographic information; 3D view; checkCIF report

## Figures and Tables

**Fig. 1.**
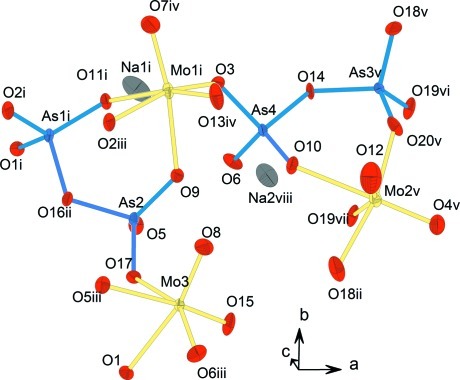
: The asymmetric unit of Na2(MoO2)3(As2O7)2. Displacement ellipsoids are drawn at the 50% probability level. symmetry codes:(i) -x + 1, y + 1/2, -z + 1/2; (ii) -x + 1, y - 1/2, -z + 1/2; (iii) x, -y + 1/2, z + 1/2; (iv) -x + 1, y + 1/2, -z + 3/2; (v) x + 1, y, z; (vi) x + 1, y, z - 1; (vii) -x + 1, -y + 1, -z + 1; (viii) -x + 1, y - 1/2, -z + 3/2.

**Fig. 2.**
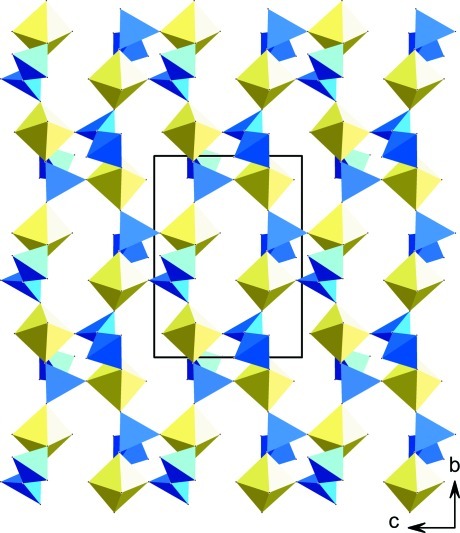
: Representation of the (Mo2O2As2O7) layer along the a axis.

**Fig. 3.**
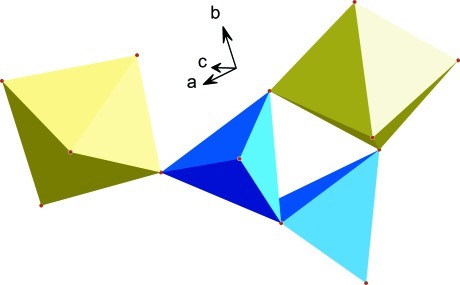
: Representation of Mo2As2O16 unit.

**Fig. 4.**
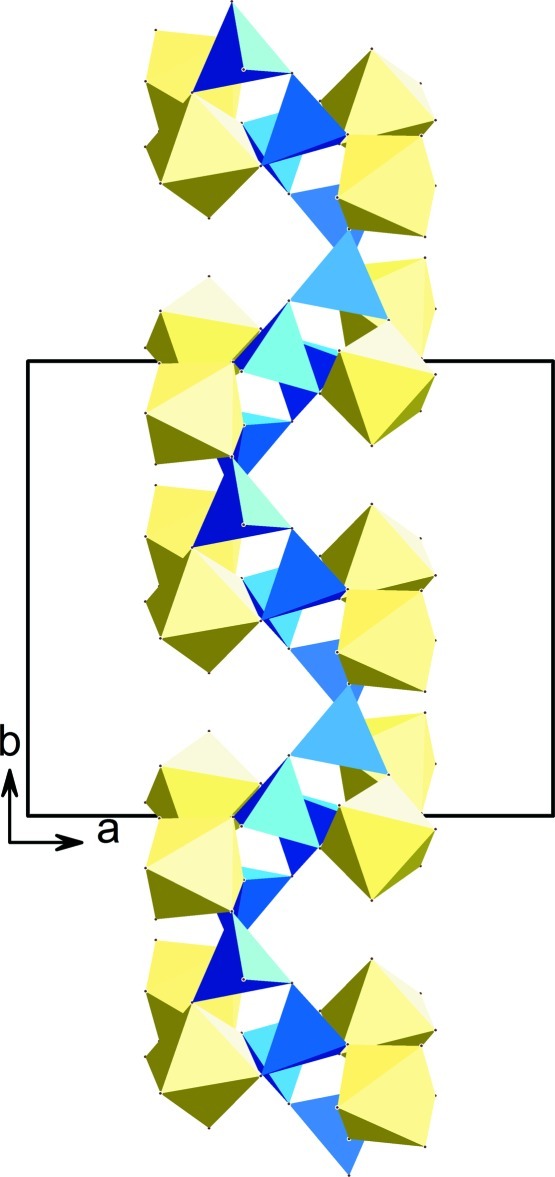
: Projection of Mo2As2O14 layer along the c axis.

**Fig. 5.**
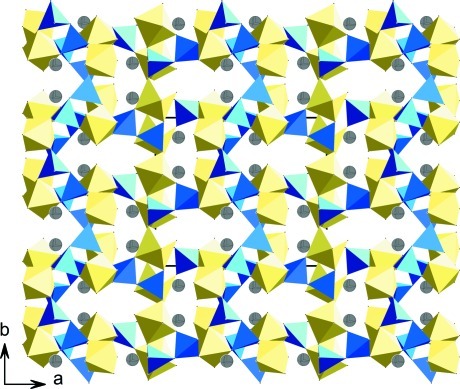
: Projection of structure according to c axis, showing the channel where Na+ cations are located.

## supplementary crystallographic information

### Comment

The search for new materials with open structure has motivated many works (Kierkegaard & Westerlund, 1964; Lii *et al.*, 1987; Guesdon *et al.*, 1994; Masquelier *et al.*, 1995). In these materials, the association of XO_4_ (*X*=P, As) tetrahedra and MO_6_ (*M*= transition metal) octahedra forms covalent hybrid structures that delimit tunnels (Linnros, 1970; Hammond & Barbier, 1996) or interlayers favorable to the migration of cations. For instance, the junction between these polyhedra can develop new materials that could have properties associated with the ion mobility of metal cations. This area is far from being fully explored and represents a field of considerable activity including several disciplines. In this context the A—Mo—As—O (A = monovalent cation) systems were explored and several interesting phases: K_2_MoO_2_As_2_O_7_ (Zid & Jouini, 1996*a*), Rb_2_MoO_2_As_2_O_7_ (Zid *et al.*, 1998) and K_2_MoO_2_(MoO_2_As_2_O_7_)_2_ (Zid & Jouini, 1996*b*) were characterized. Herein, we describe the synthesis of a new material of monoclinic symmetry by solid state reaction. The method of preparation, structure determination by X-ray diffraction on single-crystal and physical properties are presented. The new Na_2_(MoO_2_)_3_(As_2_O_7_)_2_ phase presents a three-dimensional framework. The asymmetric unit is built from two cyclic MoAs_2_O_11_ units and MoO_6_ corner-sharing octahedron (Fig. 1). The anionic framework of Na_2_(MoO_2_)_3_(As_2_O_7_)_2_ can be decomposed into two layers: the first found in the material K_2_MoO_2_(MoO_2_As_2_O_7_)_2_ (Zid & Jouini, 1996*b*), and the second Mo_2_As_2_O_14_ layer which is new. Layers (Mo2O_2_As_2_O_7_) are constructed from conventional Mo2As_2_O_11_ units in which each diarsenate group shares two oxygen atoms with the Mo2O_6_ octahedron. Units Mo2As_2_O_11_ are pairs connected to form double [Mo2_2_As_4_O_20_] units. The junction between the latter sharing corners with polyhedra of different nature, leads to the (Mo2O_2_As_2_O_7_) layer (Fig. 2) in which each Mo2O_6_ octahedron shares four of its corners with only three As_2_O_7_ groups. The other two free remaining corners form the Mo2O_2_ molybdyl group directed to large elongated parallel channels arranged to the [100] direction resulting from the combination of four double [Mo2_2_As_4_O_20_] units. The layers of the second type are built from new units of formula Mo_2_As_2_O_16_ (Fig. 3). These are due to the association between a conventional Mo1As_2_O_11_ unit and Mo3O_6_ octahedron by corner-sharing. In addition, each Mo_2_As_2_O_16_ unit binds to its centrosymmetric according to [001] direction forming double Mo_4_As_4_O_30_ units. The latter are connected by corner-sharing and form new layers of Mo_2_As_2_O_14_ type, which are organized in a wavy arrangement according to the plans (010) (Fig. 4). Within these layers there are two types of channels, hexagonal or square sections, arranged in the [010] direction. The anionic framework is constructed by the two types of layers previously described (Mo2O_2_As_2_O_7_)) (Fig. 2) and Mo_2_As_2_O_14_ (Fig. 4) using mixte Mo—O—As and As—O—Mo bridges leading to a new three-dimensional framework with large channels of two types where the Na^+^ cations are located (Fig. 5). The average Mo—O, As—O and Na—O distances in the structure are consistent with those found in the literature (Zid *et al.*, 1997; Hajji *et al.*, 2004; Hajji & Zid, 2006; Ben Hlila *et al.*, 2009). In addition, the calculation of the various valence bonds (BVS) using empirical formula of Brown (Brown & Altermatt, 1985) confirms the expected values of ion charges: Mo1(5,967), Mo2(6,017), Mo3(6,021), As1(4,966), As2(4,990), As3(5,016), As4(4,981), Na1(0,606) and Na2(0,902). The phase studied is new but the comparaison of the structure of Na_2_(MoO_2_)_3_(As_2_O_7_)_2_ with those found in the literature for related *MX*_2_O_11_ (*X*=P, As) units reveals some structural affiliation. In the diphosphates of formula A_2_MoO_2_P_2_O_7_ (A=Cs (Averbuch-Pouchot, 1988); A= NH_4_ (Averbuch-Pouchot, 1989); A=K (Zid *et al.*, 2003)), MoP_2_O_11_ units are connected by cornrer-sharing octahedra and tetrahedra to form ribbons leading to a one-dimensional framework. The combination of ribbons of type MO_2_*X*_2_O_7_ (*M*= transition metal, *X*= P, As) by M—O—*X* mixed bridges leads to layered structures similar to those encountered in diphosphates of the formula A_2_VOP_2_O_7_ (A=Rb (Lii & Wang, 1989); A=Na (Benhamada *et al.*, 1992)). The combination of such layers can lead to different three-dimensional frameworks. The structure of K_2_MoO_2_(MoO_2_As_2_O_7_)_2_ (Zid & Jouini, 1996*b*) was obtained by insertion of the MoO_2_ group between MoO_2_As_2_O_7_ layers, but in our case the two types of layers in Na_2_(MoO_2_)_3_(As_2_O_7_)_2_ are connected directly by mixed Mo—O—As and As—O—Mo bridges. In order to use the structural data obtained which are in favor of a good ion mobility and related to physicochemical properties and in particular ionic conduction, measurements of resistance as a function of temperature were carried out using an impedance analyzer of type HP4192A of a pure sample compacted in pellet and having a geometric factor of (e/s=0,017 cm^-1^). The values of the conductivities obtained by rising temperatures correlate well with the Arrhenius law: Ln(σT) = f(10^4^/T). These results show that this material has an activation energy of 0,79 eV and a conductivity of 4,294 10^-5^ S.cm^-1^ at 793 K indicating that the material can be classified as a mild ionic conductor. These results are comparable to those found in the literature for compounds with cobalt (Marzouki *et al.*, 2010) and iron (Ouerfelli *et al.*, 2007).

### Experimental

The crystals of the Na_2_(MoO_2_)_3_(As_2_O_7_)_2_ phase were obtained from the reagents: (NH_4_)_2_Mo_4_O_13_ (Fluka, 69858), NH_4_H_2_AsO_4_ (prepared in the laboratory, ASTM 01–775) and Na_2_CO_3_ (Prolabo, 27778) taken in Na: Mo: As molar ratio of 2: 3: 4. The finely ground mixture was preheated in air at 623 K to remove volatile compounds. It was then heated, in steps of 100 degrees followed by grinding to a temperature close to the melting point 828 K. The mixture was then left at this temperature for one week to promote germination and growth of crystals. The final residue was subjected to cooling first a slow (5°/half-day at 778 K) and a second faster (50°/h) to room temperature. Yellow crystals of sufficient size for the measurements of intensities, were separated from refluxing water. A qualitative analysis of a selected crystal by scanning electron microscopy (S.E.*M*) of the type FEI Quanta 200 under polarizing microscope, confirmed the presence of different chemical elements expected: As, Mo, Na and oxygen.

### Refinement

In the final refinement, examination of the Fourier-Difference reveals the presence of two peaks which are close to the oxygen atoms O19 and O20. In addition, the thermal motion of these atoms is relatively high, so each oxygen atom is distribute between two positions with variable occupancy ratios satisfying the condition of electrical neutrality of the material. For this, the use of two conditions SUMP and EADP allowed by the *SHELX* program is necessary. The ellipsoids of the atoms considered are better defined.

### Crystal data

**Table:**

Na_2_(MoO_2_)_3_(As_2_O_7_)_2_	*F*(000) = 1760
*M**_r_* = 953.48	*D*_x_ = 3.743 Mg m^−^^3^
Monoclinic, *P*2_1_/*c*	Mo *K*α radiation, λ = 0.71073 Å
Hall symbol: -P 2ybc	Cell parameters from 25 reflections
*a* = 14.571 (3) Å	θ = 10–15°
*b* = 12.580 (2) Å	µ = 10.11 mm^−^^1^
*c* = 9.258 (2) Å	*T* = 298 K
β = 94.51 (2)°	Prism, yellow
*V* = 1691.9 (6) Å^3^	0.26 × 0.18 × 0.14 mm
*Z* = 4	

### Data collection

**Table:**

Enraf–Nonius CAD-4 diffractometer	3128 reflections with *I* > 2σ(*I*)
Radiation source: fine-focus sealed tube	*R*_int_ = 0.022
Graphite monochromator	θ_max_ = 27.0°, θ_min_ = 2.1°
ω/2θ scans	*h* = −18→18
Absorption correction: ψ scan (North *et al.*, 1968)	*k* = −1→16
*T*_min_ = 0.133, *T*_max_ = 0.246	*l* = −11→1
4271 measured reflections	2 standard reflections every 120 min
3676 independent reflections	intensity decay: 1.1%

### Refinement

**Table:**

Refinement on *F*^2^	Primary atom site location: structure-invariant direct methods
Least-squares matrix: full	Secondary atom site location: difference Fourier map
*R*[*F*^2^ > 2σ(*F*^2^)] = 0.025	*w* = 1/[σ^2^(*F*_o_^2^) + (0.0188*P*)^2^ + 7.737*P*] where *P* = (*F*_o_^2^ + 2*F*_c_^2^)/3
*wR*(*F*^2^) = 0.060	(Δ/σ)_max_ = 0.001
*S* = 1.06	Δρ_max_ = 0.98 e Å^−^^3^
3676 reflections	Δρ_min_ = −0.75 e Å^−^^3^
273 parameters	Extinction correction: *SHELXL97* (Sheldrick, 2008), Fc^*^=kFc[1+0.001xFc^2^λ^3^/sin(2θ)]^-1/4^
2 restraints	Extinction coefficient: 0.00106 (6)

### Special details

**Table:**

Geometry. All e.s.d.'s (except the e.s.d. in the dihedral angle between two l.s. planes) are estimated using the full covariance matrix. The cell e.s.d.'s are taken into account individually in the estimation of e.s.d.'s in distances, angles and torsion angles; correlations between e.s.d.'s in cell parameters are only used when they are defined by crystal symmetry. An approximate (isotropic) treatment of cell e.s.d.'s is used for estimating e.s.d.'s involving l.s. planes.
Refinement. Refinement of *F*^2^ against ALL reflections. The weighted *R*-factor *wR* and goodness of fit *S* are based on *F*^2^, conventional *R*-factors *R* are based on *F*, with *F* set to zero for negative *F*^2^. The threshold expression of *F*^2^ > σ(*F*^2^) is used only for calculating *R*-factors(gt) *etc*. and is not relevant to the choice of reflections for refinement. *R*-factors based on *F*^2^ are statistically about twice as large as those based on *F*, and *R*- factors based on ALL data will be even larger.

### Fractional atomic coordinates and isotropic or equivalent isotropic displacement parameters (Å^2^)

**Table:**

	*x*	*y*	*z*	*U*_iso_*/*U*_eq_	Occ. (<1)
Mo1	0.31869 (3)	0.06243 (4)	0.05295 (4)	0.01284 (10)	
Mo2	0.02062 (3)	0.37400 (3)	0.26296 (5)	0.01470 (11)	
Mo3	0.70516 (3)	0.12821 (3)	0.42003 (4)	0.00864 (10)	
As1	0.53859 (3)	0.00042 (4)	0.20483 (5)	0.00852 (11)	
As2	0.60470 (3)	0.33825 (4)	0.24714 (5)	0.00885 (11)	
As3	0.00777 (3)	0.62745 (4)	0.12380 (6)	0.01163 (11)	
As4	0.81642 (3)	0.53068 (4)	0.18604 (5)	0.00924 (11)	
Na1	0.4171 (2)	0.1428 (2)	0.4537 (3)	0.0381 (7)	
Na2	0.13444 (18)	0.8679 (2)	0.9496 (3)	0.0343 (6)	
O1	0.6093 (2)	0.0020 (3)	0.3549 (4)	0.0129 (7)	
O2	0.5933 (2)	0.0231 (3)	0.0556 (4)	0.0146 (7)	
O3	0.7475 (2)	0.6100 (3)	0.2769 (4)	0.0150 (7)	
O4	0.1230 (3)	0.3287 (3)	0.2208 (5)	0.0291 (10)	
O5	0.5893 (2)	0.3594 (3)	0.0702 (4)	0.0163 (7)	
O6	0.7499 (3)	0.4947 (3)	0.0395 (4)	0.0189 (8)	
O7	0.3449 (3)	0.1862 (3)	0.9950 (4)	0.0276 (9)	
O8	0.7566 (3)	0.2271 (3)	0.5219 (4)	0.0225 (8)	
O9	0.6874 (2)	0.4098 (3)	0.3346 (4)	0.0136 (7)	
O10	0.8787 (2)	0.4405 (3)	0.2789 (4)	0.0161 (7)	
O11	0.4431 (2)	0.0715 (3)	0.2150 (4)	0.0125 (7)	
O12	0.0325 (3)	0.3820 (4)	0.4454 (5)	0.0425 (12)	
O13	0.2237 (3)	0.0292 (4)	0.9471 (4)	0.0259 (9)	
O14	0.8884 (2)	0.6242 (3)	0.1131 (4)	0.0149 (7)	
O15	0.7762 (2)	0.1145 (3)	0.2859 (4)	0.0222 (9)	
O16	0.5032 (2)	0.8672 (3)	0.1910 (4)	0.0123 (7)	
O17	0.6118 (2)	0.2098 (3)	0.2942 (4)	0.0147 (7)	
O18	0.0423 (3)	0.7296 (3)	0.2295 (5)	0.0366 (12)	
O19	0.0424 (7)	0.6173 (10)	0.9610 (11)	0.0191 (19)	0.612 (17)
O191	0.0395 (12)	0.6633 (14)	0.9569 (19)	0.0191 (19)	0.388 (17)
O20	0.0489 (4)	0.5281 (5)	0.2364 (9)	0.0181 (15)	0.703 (12)
O201	0.0481 (10)	0.5117 (13)	0.164 (2)	0.0181 (15)	0.298 (12)

### Atomic displacement parameters (Å^2^)

**Table:**

	*U*^11^	*U*^22^	*U*^33^	*U*^12^	*U*^13^	*U*^23^
Mo1	0.00827 (19)	0.0211 (2)	0.0093 (2)	0.00355 (16)	0.00231 (15)	0.00338 (16)
Mo2	0.0095 (2)	0.0139 (2)	0.0205 (2)	−0.00034 (16)	0.00001 (17)	0.00670 (17)
Mo3	0.00620 (19)	0.0103 (2)	0.0094 (2)	0.00053 (15)	0.00019 (14)	0.00045 (15)
As1	0.0061 (2)	0.0123 (2)	0.0073 (2)	−0.00051 (17)	0.00123 (17)	−0.00062 (17)
As2	0.0072 (2)	0.0101 (2)	0.0095 (2)	0.00085 (17)	0.00259 (17)	0.00266 (18)
As3	0.0083 (2)	0.0091 (2)	0.0182 (3)	−0.00207 (18)	0.00470 (19)	−0.00203 (19)
As4	0.0057 (2)	0.0116 (2)	0.0105 (2)	−0.00142 (17)	0.00102 (17)	−0.00197 (18)
Na1	0.0537 (17)	0.0409 (15)	0.0194 (12)	0.0219 (13)	−0.0001 (11)	−0.0080 (11)
Na2	0.0316 (14)	0.0373 (15)	0.0341 (14)	0.0110 (11)	0.0024 (11)	0.0004 (11)
O1	0.0126 (17)	0.0139 (17)	0.0115 (17)	−0.0014 (14)	−0.0039 (13)	−0.0012 (13)
O2	0.0116 (17)	0.0240 (19)	0.0091 (17)	0.0022 (14)	0.0064 (13)	0.0021 (14)
O3	0.0155 (17)	0.0130 (17)	0.0179 (18)	−0.0009 (14)	0.0091 (14)	−0.0030 (14)
O4	0.017 (2)	0.023 (2)	0.048 (3)	0.0010 (17)	0.0076 (19)	0.0048 (19)
O5	0.0149 (18)	0.0234 (19)	0.0107 (17)	−0.0006 (15)	0.0011 (14)	0.0043 (14)
O6	0.023 (2)	0.0188 (19)	0.0137 (18)	−0.0089 (15)	−0.0053 (15)	−0.0032 (15)
O7	0.028 (2)	0.027 (2)	0.029 (2)	0.0067 (18)	0.0126 (18)	0.0129 (18)
O8	0.0198 (19)	0.021 (2)	0.025 (2)	−0.0005 (15)	−0.0092 (16)	−0.0054 (16)
O9	0.0109 (16)	0.0136 (17)	0.0163 (18)	0.0005 (13)	0.0003 (14)	0.0004 (14)
O10	0.0095 (16)	0.0187 (18)	0.0203 (19)	0.0036 (14)	0.0024 (14)	0.0041 (15)
O11	0.0084 (16)	0.0156 (18)	0.0135 (17)	0.0012 (13)	0.0012 (13)	−0.0039 (14)
O12	0.029 (2)	0.068 (4)	0.029 (3)	0.002 (2)	−0.001 (2)	0.003 (2)
O13	0.0128 (18)	0.047 (3)	0.017 (2)	0.0070 (18)	−0.0025 (15)	−0.0025 (18)
O14	0.0055 (16)	0.0152 (18)	0.025 (2)	−0.0020 (13)	0.0052 (14)	0.0046 (15)
O15	0.0131 (18)	0.032 (2)	0.023 (2)	0.0060 (16)	0.0096 (15)	0.0064 (17)
O16	0.0087 (16)	0.0129 (17)	0.0159 (17)	−0.0016 (13)	0.0049 (13)	−0.0016 (13)
O17	0.0126 (17)	0.0088 (16)	0.0218 (19)	0.0015 (13)	−0.0041 (14)	0.0032 (14)
O18	0.015 (2)	0.024 (2)	0.069 (3)	0.0017 (17)	−0.004 (2)	−0.030 (2)
O19	0.012 (2)	0.030 (6)	0.017 (2)	0.006 (5)	0.0075 (17)	0.005 (5)
O191	0.012 (2)	0.030 (6)	0.017 (2)	0.006 (5)	0.0075 (17)	0.005 (5)
O20	0.013 (2)	0.019 (3)	0.021 (4)	−0.0069 (19)	−0.009 (3)	0.009 (3)
O201	0.013 (2)	0.019 (3)	0.021 (4)	−0.0069 (19)	−0.009 (3)	0.009 (3)

### Geometric parameters (Å, º)

**Table:**

Mo1—O13^i^	1.684 (4)	As2—O9	1.663 (3)
Mo1—O7^i^	1.700 (4)	As2—O17	1.675 (3)
Mo1—O3^ii^	2.002 (3)	As2—O16^ii^	1.753 (3)
Mo1—O2^iii^	2.003 (3)	As3—O19^i^	1.631 (10)
Mo1—O9^ii^	2.189 (3)	As3—O18	1.669 (4)
Mo1—O11	2.264 (3)	As3—O20	1.706 (6)
Mo2—O4	1.672 (4)	As3—O14^vi^	1.735 (3)
Mo2—O12	1.688 (5)	As4—O10	1.651 (3)
Mo2—O20	2.002 (6)	As4—O6	1.666 (3)
Mo2—O18^iv^	2.038 (4)	As4—O3	1.686 (3)
Mo2—O19^v^	2.204 (10)	As4—O14	1.747 (3)
Mo2—O10^vi^	2.246 (3)	Na1—O7^ix^	2.438 (5)
Mo3—O15	1.686 (4)	Na1—O11	2.442 (4)
Mo3—O8	1.699 (4)	Na1—O1^x^	2.592 (4)
Mo3—O6^vii^	1.982 (3)	Na1—O5^vii^	2.652 (4)
Mo3—O17	2.003 (3)	Na2—O8^xi^	2.379 (5)
Mo3—O1	2.168 (3)	Na2—O13^xii^	2.412 (5)
Mo3—O5^vii^	2.275 (4)	Na2—O20^xiii^	2.603 (8)
As1—O11	1.663 (3)	Na2—O15^xiv^	2.636 (5)
As1—O1	1.664 (3)	Na2—O18^xiii^	2.651 (6)
As1—O2	1.673 (3)	Na2—O10^xi^	2.695 (4)
As1—O16^viii^	1.755 (3)	Na2—O12^xv^	2.695 (5)
As2—O5	1.657 (3)		
			
O13^i^—Mo1—O7^i^	103.8 (2)	O11—As1—O16^viii^	105.99 (16)
O13^i^—Mo1—O3^ii^	96.13 (17)	O1—As1—O16^viii^	103.40 (16)
O7^i^—Mo1—O3^ii^	96.25 (17)	O2—As1—O16^viii^	105.00 (17)
O13^i^—Mo1—O2^iii^	95.99 (17)	O5—As2—O9	115.52 (17)
O7^i^—Mo1—O2^iii^	99.49 (17)	O5—As2—O17	114.38 (18)
O3^ii^—Mo1—O2^iii^	157.22 (14)	O9—As2—O17	111.62 (17)
O13^i^—Mo1—O9^ii^	89.84 (17)	O5—As2—O16^ii^	103.55 (17)
O7^i^—Mo1—O9^ii^	166.34 (17)	O9—As2—O16^ii^	111.25 (16)
O3^ii^—Mo1—O9^ii^	81.23 (13)	O17—As2—O16^ii^	98.86 (16)
O2^iii^—Mo1—O9^ii^	79.59 (13)	O19^i^—As3—O18	120.1 (5)
O13^i^—Mo1—O11	167.66 (18)	O19^i^—As3—O20	112.8 (5)
O7^i^—Mo1—O11	88.54 (17)	O18—As3—O20	97.5 (3)
O3^ii^—Mo1—O11	82.75 (13)	O19^i^—As3—O14^vi^	109.1 (4)
O2^iii^—Mo1—O11	81.30 (13)	O18—As3—O14^vi^	107.80 (18)
O9^ii^—Mo1—O11	77.84 (12)	O20—As3—O14^vi^	108.7 (2)
O4—Mo2—O12	103.3 (2)	O10—As4—O6	119.85 (18)
O4—Mo2—O20	96.0 (2)	O10—As4—O3	118.10 (18)
O12—Mo2—O20	93.4 (3)	O6—As4—O3	103.65 (18)
O4—Mo2—O18^iv^	96.68 (19)	O10—As4—O14	110.01 (17)
O12—Mo2—O18^iv^	91.7 (2)	O6—As4—O14	101.34 (18)
O20—Mo2—O18^iv^	164.8 (2)	O3—As4—O14	101.14 (16)
O4—Mo2—O19^v^	96.4 (3)	O7^ix^—Na1—O11	124.45 (17)
O12—Mo2—O19^v^	160.3 (3)	O7^ix^—Na1—O1^x^	115.09 (16)
O20—Mo2—O19^v^	84.9 (4)	O11—Na1—O1^x^	113.72 (15)
O18^iv^—Mo2—O19^v^	85.5 (3)	O7^ix^—Na1—O5^vii^	110.59 (16)
O4—Mo2—O10^vi^	170.19 (18)	O11—Na1—O5^vii^	98.91 (14)
O12—Mo2—O10^vi^	86.26 (19)	O1^x^—Na1—O5^vii^	84.34 (13)
O20—Mo2—O10^vi^	81.20 (19)	O8^xi^—Na2—O13^xii^	105.79 (16)
O18^iv^—Mo2—O10^vi^	84.89 (15)	O8^xi^—Na2—O20^xiii^	137.2 (2)
O19^v^—Mo2—O10^vi^	74.1 (3)	O13^xii^—Na2—O20^xiii^	78.22 (17)
O15—Mo3—O8	102.3 (2)	O8^xi^—Na2—O15^xiv^	77.62 (15)
O15—Mo3—O6^vii^	97.89 (17)	O13^xii^—Na2—O15^xiv^	67.62 (15)
O8—Mo3—O6^vii^	98.61 (17)	O20^xiii^—Na2—O15^xiv^	64.43 (18)
O15—Mo3—O17	93.01 (17)	O8^xi^—Na2—O18^xiii^	91.96 (15)
O8—Mo3—O17	101.38 (16)	O13^xii^—Na2—O18^xiii^	128.51 (16)
O6^vii^—Mo3—O17	154.59 (15)	O20^xiii^—Na2—O18^xiii^	57.74 (16)
O15—Mo3—O1	98.02 (17)	O15^xiv^—Na2—O18^xiii^	69.91 (14)
O8—Mo3—O1	159.50 (17)	O8^xi^—Na2—O10^xi^	104.17 (15)
O6^vii^—Mo3—O1	76.15 (14)	O13^xii^—Na2—O10^xi^	78.55 (14)
O17—Mo3—O1	79.65 (13)	O20^xiii^—Na2—O10^xi^	118.17 (18)
O15—Mo3—O5^vii^	169.94 (16)	O15^xiv^—Na2—O10^xi^	144.98 (16)
O8—Mo3—O5^vii^	85.81 (16)	O18^xiii^—Na2—O10^xi^	143.58 (16)
O6^vii^—Mo3—O5^vii^	86.56 (15)	O8^xi^—Na2—O12^xv^	128.48 (19)
O17—Mo3—O5^vii^	79.47 (14)	O13^xii^—Na2—O12^xv^	116.79 (18)
O1—Mo3—O5^vii^	74.19 (13)	O20^xiii^—Na2—O12^xv^	81.29 (19)
O11—As1—O1	114.32 (17)	O15^xiv^—Na2—O12^xv^	144.23 (17)
O11—As1—O2	114.23 (17)	O18^xiii^—Na2—O12^xv^	83.71 (16)
O1—As1—O2	112.56 (17)	O10^xi^—Na2—O12^xv^	60.61 (13)

Symmetry codes: (i) *x*, *y*, *z*−1; (ii) −*x*+1, *y*−1/2, −*z*+1/2; (iii) −*x*+1, −*y*, −*z*; (iv) −*x*, *y*−1/2, −*z*+1/2; (v) −*x*, −*y*+1, −*z*+1; (vi) *x*−1, *y*, *z*; (vii) *x*, −*y*+1/2, *z*+1/2; (viii) *x*, *y*−1, *z*; (ix) *x*, −*y*+1/2, *z*−1/2; (x) −*x*+1, −*y*, −*z*+1; (xi) −*x*+1, *y*+1/2, −*z*+3/2; (xii) *x*, *y*+1, *z*; (xiii) *x*, −*y*+3/2, *z*+1/2; (xiv) −*x*+1, −*y*+1, −*z*+1; (xv) −*x*, *y*+1/2, −*z*+3/2.
